# Protective effect of bergapten in acetic acid-induced colitis in rats

**DOI:** 10.1016/j.heliyon.2020.e04710

**Published:** 2020-08-15

**Authors:** Emmanuel A. Adakudugu, Elvis O. Ameyaw, Ernest Obese, Robert P. Biney, Isaac T. Henneh, Douglas B. Aidoo, Elizabeth N. Oge, Isaac Y. Attah, David D. Obiri

**Affiliations:** a Department of Pharmacology, Faculty of Pharmacy and Pharmaceutical Sciences, Kwame Nkrumah University of Science and Technology (KNUST), Kumasi, Ghana; b Department of Biomedical Sciences, School of Allied Health Sciences, College of Health and Allied Sciences, University of Cape Coast, Cape Coast, Ghana; c Department of Pharmacology, School of Medical Sciences, College of Health and Allied Sciences, University of Cape Coast, Cape Coast, Ghana; d School of Pharmacy and Pharmaceutical Sciences, College of Health and Allied Sciences, University of Cape Coast, Cape Coast, Ghana

**Keywords:** Pharmaceutical science, Immunology, Pathology, Physiology, Bergapten, Ulcerative colitis, Furanocoumarin, Mast cells, Immune modulators, Biological sciences, Health sciences, Gastrointestinal system, Pharmacology, Evidence-based medicine

## Abstract

Bergapten (5-methoxysporalen) is a furanocoumarin extracted from several species of citrus and bergamot oil. Bergamot essential oil is used traditionally in the management of inflammatory conditions. Previous studies on bergapten have explored mainly its *in vitro* anti-inflammatory activities which include suppression of the expression and release of pro-inflammatory cytokines such as TNF-α and interleukins as well as prostaglandins. Bergapten enhances the clearance of neutrophils and macrophages from the site of inflammation and reduces oxidative stress by inhibition of reactive oxygen species (ROS). Bergapten was assessed for its anti-inflammatory properties in acetic acid-induced colitis. Animals were obtained and randomly placed in six (6) groups (n = 5) after acclimatization. Colitis was induced by rectal administration using 4% ^v^/_v_ acetic acid in Sprague Dawley rats after pre-treatment for 5 days. Bergapten was administered at doses of 3, 10, and 30 mg kg^−1^*p.o*. while the control group received saline 5 mL kg^−1^*p.o.* and the standard drug employed was sulphasalazine at a dose of 500 mg kg^−1^. Assessments made for colon-weight-to-length ratio, colonic injury, and mucosal mast cell degranulation. There were reduced colon-weight-to-length ratios in animals treated with bergapten which was significant (*p < 0.5*) for doses 10 and 30 mg kg^−1^ compared to the disease control group Both macroscopic and microscopic damage were reduced as well, with a lesser percentage of degranulated mast cells. Macroscopic damage was reduced for bergapten at doses 10 and 30 mg kg^−1^ significantly at *p < 0.5* and *p < 0.001,* respectively. Similarly, microscopic damage was reduced at *p < 0.01* and *p < 0.001* respectively for bergapten 10 and 30 mg kg^−1^. The reduction of degranulation by bergapten was significant at *p < 0.001*. There was generally reduced damage at inflammatory sites as well as decreased infiltration of inflammatory cells. Overall, bergapten reduces inflammation in acetic acid-induced colitis.

## 1 Introduction

An inflammatory response serves to protect the body against infection, as well as enhance healing, and repair following an injury to the cells or tissues [1]. Although inflammation is a protective mechanism, mediators released during the response may lead to tissue damage. Therefore, when this system is compromised (may be due to an underlying condition or medication) this leads to persistent and opportunistic infections which increases the risk of developing cancer. On the converse, incorrect and persistent activation of inflammatory mechanisms can result in chronic inflammation. This situation is observed in several organs in the form of allergic and autoimmune diseases, including inflammatory bowel disease [2]. The constant exposure to ever-present pathogens, harmful chemicals, and physical injuries emphasizes the significance of the inflammatory reaction. The inflammatory mechanism comprises both an innate non-specific response and a more specific adaptive immune mechanism. This mechanism involves a complex cascade of cell signaling processes and encompasses several inflammatory mediators which are targets for drug action [3].

Macrophage mediators (M1 and M2) play significant roles in the process of tissue repair and mediating autoimmune diseases. M1 macrophages initiate inflammation and mediate the process of tissue repair [4, 5, 6]. M2 macrophages are responsible for phagocytosis of debris and dead cells and apoptotic neutrophils in the resolution of inflammation. The anti-inflammatory cytokines IL-10 and TGF-β M2 which are involved in tissue resolution are derived from M2 macrophages [5, 6, 7, 8]. Macrophages again are an important source of mediators in autoimmune diseases such as inflammatory bowel disease (IBD) [9, 10]. In IBD the mediators expressed from macrophages that have been identified are TNF-α, IL-1β, IL-12, IL-18, and IL-23 [9, 11, 12].

Inflammatory bowel disease (IBD) is a chronic, recurrent, and progressive disease, and there is no treatment to date that would lead to a permanent cure. Ulcerative colitis is one of the two main forms of inflammatory bowel disease (IBD) and affects mainly the colon and rectum. Its prevalence has increased in most regions of the world [1], thus creating the need for more treatment options [14]. It is characterized by continuous inflammation and ulceration of the mucosa. It affects several people globally, occurring equally among men and women. The precise cause of the disease is unknown [14, 15]. Evidence suggests a genetic predisposition to the disease [16]. Colitis is characterized by extensive epithelial cell damage, infiltration of neutrophils, and formation of crypt abscesses.

Ulcerative colitis exhibits signs of both Th1 mediated cell effects and Th2 humoral responses. Th2 responses from evidence presume to dominate. Several other cytokines are involved in the pathogenesis of the disease such as interleukins (IL)-6 and IL-1β, interferon-γ [17, 18, 19, 20]. Tumor necrosis factor (TNF-α) plays a role in the disease but is not a specific marker of the disease. Oxidative stress appears to be a significant feature in ulcerative colitis which is secondary to inflammation. Reactive oxygen species (ROS) are usually present in colons as a result of inducible nitric oxide synthase (iNOS) and cyclooxygenase-2 (COX-2) activity. Nuclear factor-kB (NF-kB) induces the production of COX-2 and hence plays a role in the pathogenesis of the disease. Current management targets inflammation by the use of anti-inflammatory agents and the reduction of oxidative stress. Acetic acid-induced colitis is frequently used in experimental studies to test new concepts and drugs potentially useful in IBD therapy [21].

The use of current anti-inflammatory drugs causes serious side effects such as ulcers and interactions with other medications in therapy [22]. As a result, there is a limitation in their use, especially in the management of chronic conditions such as colitis. Nature in its diversity provides several sources for extraction, identification, and isolation of novel compounds which may be of benefit in curbing several diseases [23]. An example of a compound showing promise as an anti-inflammatory agent is bergapten [24]. Bergapten (5-methoxysporalen) is a furocoumarin isolated from bergamot essential oil and other essential oils derived from citrus species such as *Ficus exasperata* [25]. Traditionally in Turkish medicine, bergamot essential oil is employed in the treatment of inflammation as well as in pain management and as an appetite stimulant [25]. Bergapten is used in the clinical management of psoriasis, like other psoralens [12, 25]. It is also used to manage other skin conditions such as eczema and dermatitis including skin depigmentation conditions such as leprosy, vitiligo, and leukoderma [13, 26]. Several studies have been conducted to elucidate bergapten's anti-inflammatory action.

Bergapten suppresses the production of proinflammatory cytokines; tumour necrosis factor-alpha (TNF-α) and interleukin 6 (IL-6) as shown by Bose et al. [27]. In another study by Zhou et al., (2017) [28], bergapten was shown to suppress the activation of pro-inflammatory cytokines TNF-α and IL-6 by lipopolysaccharide as demonstrated previously and further repressed prostaglandin E_2_ (PGE_2_), IL-1β, inducible nitric oxide synthase (iNOS), nitric oxide (NO), and cyclooxygenase-2 (COX-2). Bergapten again boosted the release of IL-10 in a dose dependent manner in RAW264.7 cells and the accumulation of ROS was prevented in an antioxidant assay. The recruitment of neutrophils and macrophages to the site of inflammation was reduced in a study conducted by Yang *et al.,* (2018) [29]. Due to these anti-inflammatory properties of bergapten, its effect in acetic acid-induced colitis was investigated.

## 2 Materials

### 2.1 Animals

Sprague Dawley rats of both sexes weighing between 150 ± 50 g were obtained from Noguchi Memorial Institute for Medical Research, Legon, Ghana. A total of 30 animals were used for the experiment which included 15 male and female animals each. Male and female animals were kept separate throughout the studies. They were left to acclimatize in the Animal House of the Department of Pharmacology, Kwame Nkrumah University of Science and Technology (KNUST), Ghana, one week before the study was commenced. Softwood shavings were used as bedding in the cages rats were kept in. Animals were fed with standard feed and distilled water *ad libitum* under standard housing conditions. Animals were handled in accordance with Animal Welfare Regulations (USDA 1985; US Code, 42 USC § 289d) and Guide for the Care and Use of Laboratory Animals (Institute for Laboratory Animal Research, 2011). The Ethics Committee of the Department of Pharmacology, KNUST approved all procedures used in this study.

### 2.2 Drugs and chemicals

Bergapten was acquired from Sigma Aldrich (St. Louis, USA). Sulphasalazine was obtained from Pfizer (Hampshire, England, UK). Glacial acetic acid, diethyl ether, Tween 20, chloroform, DMSO, and ethanol were obtained from VWR International (Fontenay-sous-Bois, France).

## 3 Methods

### 3.1 Induction of colitis

Rats were randomized into 6 groups (n = 5) in a stratified manner to ensure adequate distribution of male and female rats. Animals received either normal saline 5 mL kg^−1^ *p.o.*, sulphasalazine 500 mg kg^−1^ *p.o.* or bergapten 3, 10, and 30 mg kg^−1^ *p.o.* daily as a single dose for 5 days. Bergapten was prepared by solubilizing it using Tween 20 and distilled water to prepare suitable concentrations such that doses administered did not exceed 1 mL. After treatment, animals were fasted for 24 h before the induction by rectal administration of 1 mL 4% ^v^/_v_ acetic acid on day 6 [30]. The naïve group received rectal administration of distilled water. Bowel emptying was induced by administering 1 mL normal saline rectally after rats were anesthetized using diethyl ether before induction of colitis. After 24 h, animals were euthanized using chloroform and colons harvested. Assessments were made for colon weight-to-length ratio, examination for macroscopic damage, tissue histology, and mast cell count.

### 3.2 Colon weight-to-length ratio

The abdomens of rats were dissected, and colons excised by tracing backward. Colon lengths measuring 10 cm were cut out and opened longitudinally and rinsed in saline and the wet weights obtained. The weight-to-length ratio was then calculated by dividing the wet weights by the length of the colon to obtain an index of disease-caused edema and wall thickening [31].

### 3.3 Determination of extent of macroscopic damage

The extent of macroscopic damage was assessed by assigning scores as described by Millar [32]. The damage to colons was based on observed clinical features and scores assigned accordingly; no macroscopic alteration (score 0), mucosal erythema only (score 1), mild mucosal edema, slight bleeding or small erosions (score 2), moderate edema, slight bleeding ulcers or erosions (score 3) and severe ulceration, edema and tissue necrosis (score 4). These scores were assigned blindly after careful observation of colons.

### 3.4 Tissue histopathology

Sections of the harvested colons were fixed using 5 % formalin and processed for histopathological analysis. The segments were fixed in paraffin and transverse sections of 5 μm cut out using a microtome. The excised tissues were processed, mounted on slides, and tissue staining was done with hematoxylin and eosin (H & E). The slides were then viewed using a light microscope (Olympus®) fitted with a digital camera (AmScope ®). Images were obtained and assessed for histopathological damage using a cumulative semi-quantitative scale of 0–11 [33]. The quantification was done as follows: loss of mucosal architecture (0–3), cellular infiltration (0–3) muscle thickening (0–3), crypt abscess formation (0–1), and goblet cell depletion (0–1). For scales (0–3), absent (0), mild (1), moderate (2), and severe (3). And for scores (0–1), absent (0), and present (1).

### 3.5 Mast cell count

To determine the mast cell count, sections of the colons were fixed using Carnoy's fixative and entrenched in paraffin. Slices of thickness 5μm were obtained by transverse sectioning. These were placed on a slide and stained with 1 % toluidine blue (pH 4.0). The slides were then observed under a light microscope (Olympus®) fitted with a camera (AmScope®). Counts were made for both intact and degranulated cells from 5 random fields of view for each treatment group.

### 3.6 Statistical analysis

Data was analyzed using GraphPad Prism Software for Microsoft Windows, version 7.0 and results presented as mean ± SEM. One-way analysis of variance (ANOVA) was used to determine significance between group treatments with Dunnett's post hoc test at *p < 0.05*. Two-way ANOVA was employed in analyzing the time course curves followed by appropriate post hoc test.

## 4 Results

### 4.1 Colon weight-to-length ratio

**Figure 1:**
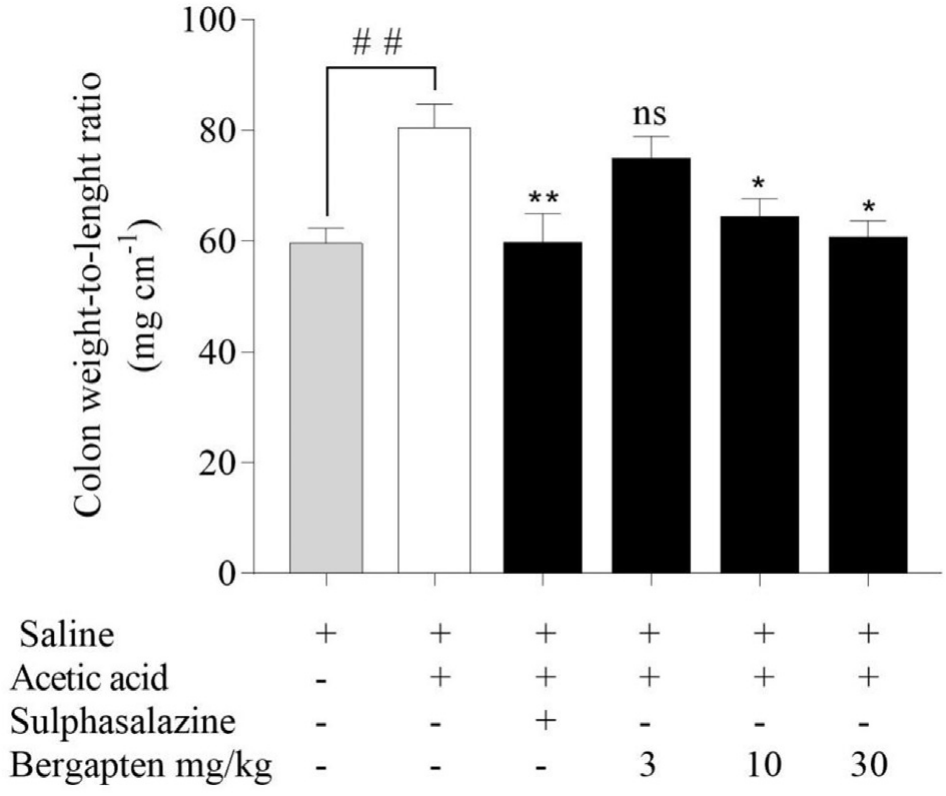
Effect of bergapten on rat colon weight-to-length ratio in acetic acid-induced colitis. Rats (n = 5) received either normal saline 5 mL kg^−1^, sulphasalazine 500 mg kg^−1^ or bergapten 3–30 mg kg^−1^ for 5 days. Colitis was induced with 4% acetic acid on the 6^th^ day after which rats were sacrificed 24 h later. Rat colons were excised, measured, and weighed. Data is presented as Mean ± S.E.M. (n = 5). ∗*p < 0.05*, *∗∗p < 0.01* compared to the acetic acid control group; ^*##*^*p < 0.01* compared to the naïve control group (One-way ANOVA followed by Dunnett's *post hoc* test). ns - not statistically different.

Rat colon weight-to-length (mg cm^−1^) was determined for all groups and the means determined. The weight-to-length ratio of the naïve control was 59.6 ± 2.7 which increased considerably to 80.4 ± 4.3 in the acetic acid disease-control (Figure 1). However, this was reduced to 59.2 ± 7.8 in the sulphasalazine treated animals. Animals that received bergapten at 3 mg kg^−1^ with a ratio of 74.3 ± 4.7 was not significant when compared to the colitic control group. Bergapten 10 mg kg^−1^ and 30 mg kg^−1^ treated rats, however, had statistically significant mean weight-to-length ratios of 64.0 ± 3.7 and 60.3 ± 3.4 respectively (Figure 1).

### 4.2 Macroscopic damage to rat colon

**Figure 2:**
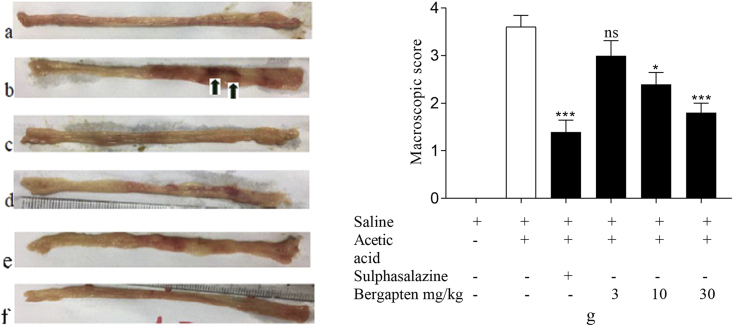
Effect of bergapten on acetic acid-induced macroscopic damage on rat colon. Sprague Dawley rats (n = 5) were treated with either normal saline 5 mL kg^−1^, sulphasalazine 500 mg kg^−1,^ or bergapten 3–30 mg kg^−1^ prophylactically for 5 days and colitis was induced with 4% acetic acid on day 6. Animals were sacrificed 24 h later and colons harvested. (a) – naive control, (b) – disease control, (c) – sulphasalazine 500 mg kg^−1^, (d–f) - bergapten 3, 10, 30 mg kg^−1^ respectively. Macroscopic damage was quantified (g). Data is presented as Mean ± S.E.M. (n = 5). ∗*p < 0.05,* ∗∗∗*p < 0.001* compared to the acetic acid control group; ^*####*^*p < 0.0001* compared to the naïve control group (One-way ANOVA followed by Dunnett's *post hoc* test). ns - not statistically different.

Harvested colons were opened longitudinally and washed in normal saline after which they were examined. After assessing colons, the naive control group showed intact mucosa and serosa with no signs of tissue damage or haemorrhage (Figure 2(a)). The acetic acid disease-control group upon examination showed extensive necrosis of tissue over a wide surface area with severe haemorrhage (Figure 2(b)). The mucosal lining was damaged with visible erosions occurring (green arrows). Sulphasalazine protected against mucosal damage and tissue necrosis. There was minimal evidence of haemorrhage (Figure 2(c)). Treatment groups that received bergapten 3–30 mg kg^−1^ did not show extensive damage to rat colons and there were no erosions on the tissues (Figures 2(d-f)). Macroscopic scores were assigned to numerically quantify tissue damage. The highest score was recorded in the disease-control group with an average score of 3.8 ± 0.4 compared to the naive control group with a score of 0 (Figure 2(g)). Sulphasalazine-treated rats recorded the lowest macroscopic score of 1.4 ± 0.3 (*p < 0.01*) while the highest dose of bergapten 30 mg kg^−1^ had a score of 1.8 ± 0.2 significant at *p < 0.05* (Figure 2(g)). Bergapten 3 mg kg^−1^ and 10 mg kg^−1^ treated rats had scores of 3 ± 0.3 and 2.6 ± 0.4 respectively (Figure 2(g)) with bergapten 10 mg kg^−1^ significant at *p < 0.05*.

### 4.3 Histopathological evaluation of rat colons

**Figure 3:**
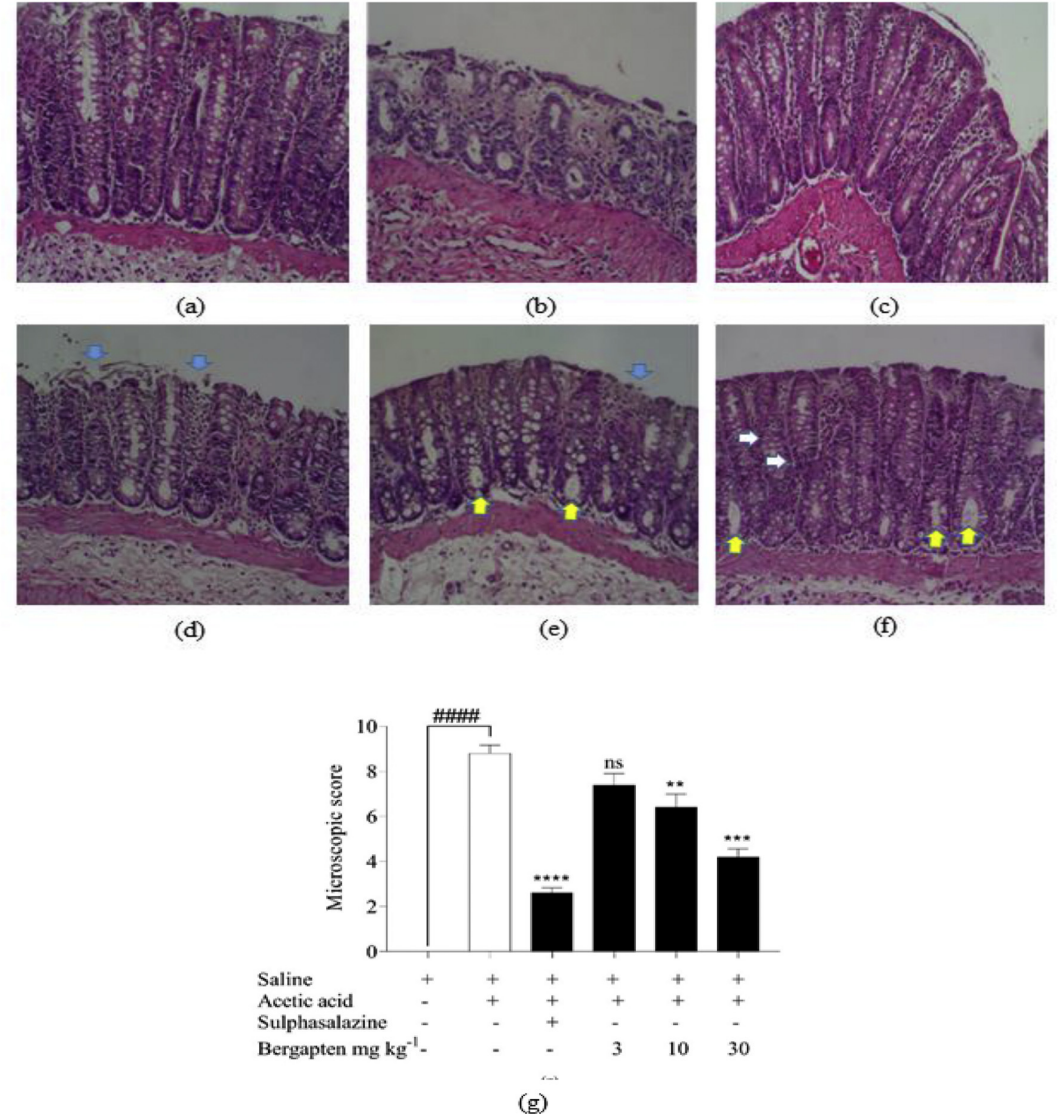
Colon histology in acetic acid-induced colitis in Sprague Dawley rats. Rats received either normal saline 5 mL kg^−1^, sulphasalazine 500 mg kg^−1^ or bergapten 3, 10, and 30 mg kg ^−1^*p.o*. Acetic acid 4% was used to induce colitis in rats. Longitudinal sections stained with H & E (a) – naive control, (b) – colitic control, (c) – Sulphasalazine 500 mg kg ^−1^, (d–f) bergapten 3, 10, 30 mg kg ^−1^ respectively. Colonic damage was quantified for the extent of injury (B). Data is presented as Mean ± S.E.M. (n = 5). ∗∗*p < 0.01,* ∗∗∗*p < 0.001,* ∗∗∗∗*p < 0.0001* compared to the acetic acid control group (AA); ^*####*^*p < 0.0001* compared to the saline-treated naïve control group (One-way ANOVA followed by Dunnett's *post hoc* test). Magnification x 40.

Rat colons were fixed in paraffin, sectioned, and stained with H & E to enable viewing under a light microscope for histopathological assessment. The naïve control group displayed normal histopathological structures and cells (Figure 3(a)). There was a regular arrangement in cell architecture with normally distributed goblet cells and visible crypts of lieberkühn. All layers; muscularis mucosae, submucosa, and muscularis externa showed homogeneity. The acetic acid disease-control group showed evident contrast to the naive group, displaying disease pathologies and tissue damage (Figure 3(b)). The epithelial lining was almost completely sloughed off (green arrows) and with irregular arrangement of cells. Goblet cells were significantly reduced and leucocytosis was increased. Sulphasalazine, however, ameliorated microscopic damage and maintained the regular arrangement of cell structure (Figure 3(c)). Goblet cells and crypts of lieberkühn were both visible and normally distributed. Leucocytosis was reduced. Groups that received bergapten 3–30 mg kg^−1^ had reduced damage to colons when compared to the disease control group (Figures 3(d–f)). Bergapten treated rats had a significant preservative effect against colonic microscopic damage, maintaining normal arrangement and distribution of cells (white arrows), there was less sloughing of the epithelial layer (blue arrows) and visible crypts of lieberkühn (yellow arrows). Microscopic scores were assigned based on the extent to colon damage on a scale of 0–11. Acetic acid disease control animals had an average microscopic score of 8.8 ± 0.4 which was reduced significantly in the sulphasalazine group to 1.4 ± 0.3. Bergapten treated rats at doses of 3, 10 and 30 mg kg^−1^ had average microscopic scores of 6.2 ± 0.6, 3.4 ± 0.5, and 1.6 ± 0.3 respectively (Figure 3(g)) which were significantly different from the disease control group.

### 4.4 Effect of bergapten on mucosal mast cell count

**Figure 4:**
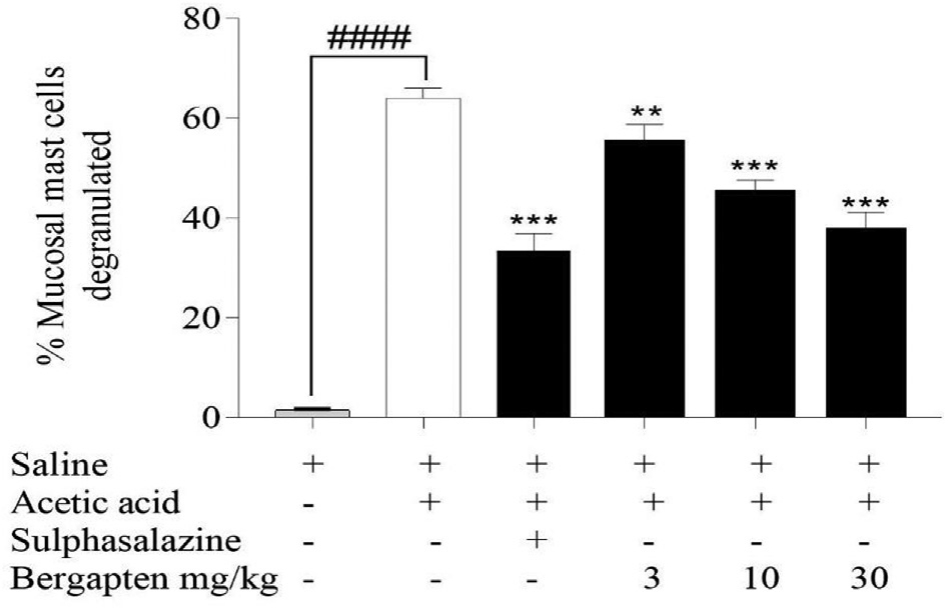
Effect bergapten on acetic acid-induced mucosal mast cell degranulation. Sprague Dawley rats (n = 5) were treated either with normal saline 5 mL kg^−1^, sulphasalazine 500 mg kg^−1,^ or bergapten 3–30 mg kg^−1^ prophylactically for 5 days and colitis induced with 4% ^v^/_v_ acetic acid on day 6. Animals were sacrificed 24 h after and colons harvested. Sections were stained with toluidine blue and viewed under a microscope (x40). A mast cell count was made and the percentage degranulated mast cells calculated. Data is presented as Mean ± S.E.M. (n = 5). ∗∗*p < 0.01,* ∗∗∗*p < 0.001* compared to the acetic acid control group; ^*####*^*p < 0.0001* compared to the saline-treated naïve group (One-way ANOVA followed by Dunnett's *post hoc* test). ns not statistically different.

Mast cells present on the mucous of the colons which were stained by toluidine blue were counted and the percentage of degranulated cells calculated. The results showed that the acetic acid disease control group had a higher percentage of mast cells being degranulated (64 ± 0.89%) while the sulphasalazine-treated group recorded a lower percentage of degranulated cells (33.4 ± 1.57%) amongst the treated groups as shown in Figure 4. Bergapten (3–30 mg kg^−1^) reduced the percentage of degranulated mast cells significantly compared to the acetic acid disease control group with mean percentages of 55 ± 1.67% at *p < 0.01*, 45 ± 1.18% at *p < 0.001* and 37.4 ± 1.69% *p < 0.001* respectively.

## 5 Discussion

Inflammation is characterized, among others by pain and edema. Other classical signs of inflammation are heat, redness and loss of function. It aids in tissue repair and helps protect the body. It may manifest in other varied forms and presents with other symptoms in different disease conditions depending on the part affected. Colitis, asthma, and arthritis are examples of chronic inflammatory conditions and this study sought to determine the effects of bergapten on acetic acid-induced colitis using murine models. Bergapten was shown to have a dose-dependent effect in ameliorating colonic damage and enhancing wound healing.

In assessing the colon, there was a significant reduction in the weight-to-length ratio. An increase in cell infiltration and edema often corresponds to an increase in colon weight in relation to the length of tissue. The reduced weight-to-length ratio may signify less active inflammation due to effective anti-inflammation therapy. Inflammatory cells contribute to edema. It is established that bergapten enhances the clearance of inflammatory cells at the site of inflammation [27] and is anticipated that clearance by bergapten may also account for the reduced colon weights. This is, however, a preliminary parameter and more accurate conclusions cannot be drawn.

Macroscopic damage was also evaluated by visualizing colons. Observed features included ulcerations and erosions in the colon. Acetic acid induces colonic damage such as ulcers, reddening, and bleeding. Bergapten enhanced wound healing and reversed macroscopic damage to colons in treated groups. Persistent inflammation may cause damage to tissues around the site of inflammation which may, in turn, further increase the inflammatory response through the production of pro-inflammatory cytokines such as TNF-α and IL-6 which are responsible for the major signs and symptoms of the disease [34]. Oxidative stress also to a large extent is a major contributory factor to the disease state of ulcerative colitis [35]. Bergapten reduces the expression of cytokines, iNOS, and prostaglandin E_2_ [28], which may contribute to the overall protective effect exhibited. Also, its antioxidative effect in reducing ROS may enhance its anti-inflammatory effect and enhance protection against damage to colons.

After the macroscopic assessment, tissues were also examined microscopically to determine cell structural changes and other associated pathologies. The colitic control exhibited sloughed off epithelial layer whiles bergapten treated colons had less epithelial damage and sloughing off. The epithelial layer is protected by mucous secreted by cells below it which also maintains it. Loss of the epithelial layer results from damage caused by inflammatory cells such as tissue macrophages. There was a reduced monocyte infiltration in the bergapten-treated group. This could be attributed to increased clearance of inflammatory cells (macrophages and neutrophils) and reduced expression of pro-inflammatory cytokines as established from previous studies by Bose [27] and Zhou [28]. Enhanced mucosal blood flow aids in protecting tissues and organs against damage. Sørbye & Svanes (1994) [36] have demonstrated that improved gastric mucosal blood flow leads to little or no damage when exposed to injurious factors while reduced blood flow increases the sensitivity of the mucosa. This would increase the extent and degree of damage to the gastric mucosa. Similar protective effects have been demonstrated in other organs such as the duodenum [37], pancreas [38], and colon [39, 40].

Reduced damage may also be attributed to the antioxidant effect of bergapten, which reduces the expression of ROS [28]. Oxidative stress contributes to a large extent the disease pathology of colitis. ROS by increasing oxidative stress leads to damage to the epithelium of colons. Bergapten has scavenging capacity on mixed reactive oxygen and nitrogen species R(N)OS (nitro oxidative stress) which would contribute to reduced sloughing off of the epithelial layer of the cell wall in colons of rats treated with bergapten. Though these mixed-species are short-lived, they cause damage to lipids and proteins and produce harmful secondary metabolites that contribute to the pathogenesis of diseases including inflammatory diseases. The damage caused by R(N)OS usually leads to the infiltration of inflammatory cells such as monocytes and lymphocytes to the site of inflammation [41]. Therefore, the reduced expression of R(N)OS may ultimately lead to reduced damage and inflammation.

Mast cells are inflammatory cells that appear as either intact or degranulated. They are degranulated when triggered and subsequently release several inflammatory mediators. These cells contribute to the disease state in ulcerative colitis and are usually more expressed in inflamed colons [42]. The rate of degranulation can be correlated to the extent of damage [43]. Mast cells were generally less degranulated at a higher dose of bergapten and this may be a result of enhanced stability of mast cells by bergapten which reduces the expression of pro-inflammatory cytokines through the blockade of IkB kinase (inhibitor of kappa B) nuclear factor-kB (Nf-kB) pathway [44] and suppression of the initiation of phosphoinositide 3-kinase (PI3K/AKT), c-Jun N-terminal kinase JNK/mitogen-activated protein kinase (MAPK) and NF-κB signalling pathways [28] These likely contributed to the overall mast cell stabilising ability of bergapten and hence reducing the percentage of degranulated cells.

Bergapten is a coumarin (furanocoumarin) [27]. Acenocoumarol and warfarin are other examples of coumarins that antagonize the effects of vitamin K and thus exhibit anticoagulant effects by interfering with the cyclic interconversion of vitamin K and its 2,3 epoxide [45]. There is a close relationship exhibited between inflammation and coagulation [46, 47] where inflammation plays a critical role in the activation of coagulation cascade and coagulation, in turn, stimulates inflammatory processes. Prior studies conducted in the gut have shown that acenocoumarol, inhibits the development of acute pancreatitis [48, 49] and enhances recovery in this disease [50]. Maduzia et al., (2020) [51], in their study also showed a similar protective effect exhibited by warfarin treatment before the induction of acute pancreatitis in the model employed in the study. Anti-inflammatory, protective and therapeutic effects have been observed in other studies on colons on the administration of coumarins such as daphnetin [52], 4-methylesculin [53], or osthole [54]. Although studies have not been done on the anticoagulant effect of bergapten, results from these experiments conducted indicate that possible, anti-inflammatory action shown by bergapten may be due to its possible anticoagulant activity.

## 6 Conclusion

Bergapten reduces inflammation in acetic acid-induced inflammation in rats, reducing damage to colons and mast cell degranulation. Its use in the management of the condition should be further explored as it may provide an alternative to treatment in the future.

## Declarations

### Author contribution statement

E.O. Ameyaw, R.P. Biney: Conceived and designed the experiments; Analyzed and interpreted the data.

E.A. Adakudugu: Performed the experiments; Contributed reagents, materials, analysis tools or data; Wrote the paper.

E. Obese and D.D. Obiri: Conceived and designed the experiments; Contributed reagents, materials, analysis tools or data.

I.T. Henneh: Performed the experiments.

D.B. Aidoo: Performed the experiments; Contributed reagents, materials, analysis tools or data.

E.N. Oge: Performed the experiments; Wrote the paper.

I.Y. Attah: Analyzed and interpreted the data; Wrote the paper.

### Funding statement

This research did not receive any specific grant from funding agencies in the public, commercial, or not-for-profit sectors.

### Competing interest statement

The authors declare no conflict of interest.

### Additional information

No additional information is available for this paper.
